# Ischemic damage to every segment of the oxidative phosphorylation cascade elevates ETC driving force and ROS production in cardiac mitochondria

**DOI:** 10.1152/ajpheart.00129.2022

**Published:** 2022-07-22

**Authors:** Sarah Kuzmiak-Glancy, Brian Glancy, Matthew W. Kay

**Affiliations:** ^1^Department of Kinesiology, School of Public Health, University of Maryland, College Park, Maryland; ^2^Laboratory of Muscle Energetics, National Heart, Lung, and Blood Institute and National Institute of Arthritis and Musculoskeletal and Skin Diseases, National Institutes of Health, Bethesda, Maryland; ^3^Department of Biomedical Engineering, The George Washington University, Washington, District of Columbia

**Keywords:** cardiac ischemia, metabolic control, mitochondria, reactive oxygen species

## Abstract

Myocardial ischemia has long-lasting negative impacts on cardiomyocyte mitochondrial ATP production. However, the location(s) of damage to the oxidative phosphorylation pathway responsible for altered mitochondrial function is unclear. Mitochondrial reactive oxygen species (ROS) production increases following ischemia, but the specific factors controlling this increase are unknown. To determine how ischemia affects the mitochondrial energy conversion cascade and ROS production, mitochondrial driving forces [redox potential and membrane potential (ΔΨ)] were measured at resting, intermediate, and maximal respiration rates in mitochondria isolated from rat hearts after 60 min of control flow (control) or no-flow ischemia (ischemia). The effective activities of the dehydrogenase enzymes, the electron transport chain (ETC), and ATP synthesis and transport were computed using the driving forces and flux. Ischemia lowered maximal mitochondrial respiration rates and diminished the responsiveness of respiration to both redox potential and ΔΨ. Ischemia decreased the activities of every component of the oxidative phosphorylation pathway: the dehydrogenase enzymes, the ETC, and ATP synthesis and transport. ROS production was linearly related to driving force down the ETC; however, ischemia mitochondria demonstrated a greater driving force down the ETC and higher ROS production. Overall, results indicate that ischemia ubiquitously damages the oxidative phosphorylation pathway, reduces mitochondrial sensitivity to driving forces, and augments the propensity for electrons to leak from the ETC. These findings underscore that strategies to improve mitochondrial function following ischemia must target the entire mitochondrial energy conversion cascade.

**NEW & NOTEWORTHY** This integrative analysis is the first to assess how myocardial ischemia alters the mitochondrial driving forces and the degree to which individual segments of the mitochondrial energy transduction pathway contribute to diminished function following ischemia. This investigation demonstrates that increased reactive oxygen species production following ischemia is related to a lower effective activity of the electron transport chain and a greater driving force down the electron transport chain.

## INTRODUCTION

The heart is critically dependent on oxygen and mitochondrial metabolism for ATP production. When myocardial blood flow is limited, as occurs during ischemia, there are long-lasting negative impacts on mitochondrial oxidative phosphorylation. Previous investigations demonstrate that mitochondria isolated from ischemic cardiac tissue have reduced maximal oxygen consumption rates and increased reactive oxygen species (ROS) production (1–8). However, the locations and degree of damage to the oxidative phosphorylation pathway during ischemia remain unknown, as well as how the damage and the resultant altered electrochemical gradients contribute to increased ROS production after ischemia.

The conversion of mitochondrial fuels into ATP requires the proper functioning of the mitochondrial fuel transporters, dehydrogenase enzymes, electron transport chain (ETC), ATP synthase, and the adenine nucleotide translocase (ANT). Each of these components contribute to the control of cellular oxidative phosphorylation (9–13) and damage to any of these components could cause the altered mitochondrial function observed following ischemia. Indeed, investigations have implicated altered activities of the ETC (7, 8, 14), ATP synthase (15), and ANT after ischemia (16, 17). The studies mentioned above isolated mitochondria from previously ischemic myocardial tissue and individually assessed the role of the specific component of interest. Indeed, the component that investigators assessed was concluded to be responsible, at least in part, for altered mitochondrial function following ischemia (7, 8, 14–19). Furthermore, a majority of these studies isolated a specific component of interest and assessed the activity of that component using nonphysiological, nonphosphorylating conditions, such as assessing isolated complex I activity in the absence of mitochondrial membrane potential. Finally, there is a lack of information regarding how an episode of ischemia alters the ability of mitochondrial dehydrogenase enzymes to harvest electrons from fuel, as none of the aforementioned studies measured changes in dehydrogenase activity.

A primary objective of the present study was to measure the oxygen consumption of intact, respiring mitochondria isolated from normoxic and previously ischemic ventricular tissue to determine the effective activities of *1*) the fuel transporters and dehydrogenase enzymes; *2*) the ETC; and *3*) ATP synthesis and transport (ATP synthase/ANT/P_i_ transporter). Mitochondrial driving forces [oxidation-reduction (redox) potential and membrane potential (ΔΨ)] were measured while varying the ADP:ATP ratio to produce intermediate oxygen consumption rates. This was accomplished using the progressive creatine kinase energy clamp technique, which stepwise decreases the ADP:ATP ratio, altering Gibbs-free energy of ATP (ΔG_ATP_), and assesses the ability of the mitochondria to respond to the changing energetic status (20–23).

Assessments of ROS production from mitochondria isolated from previously ischemic myocardial tissue have consistently shown higher rates of ROS release (7, 8, 18, 19). It is common, when assessing ROS production, to use conditions that elicit maximal ROS production. This includes assessments of ROS production *1*) under nonphosphorylating (low respiration) conditions, when mitochondrial redox potential and membrane potential are maximally reduced/polarized and *2*) using inhibitors of the respiratory chain and ATP synthase to maximize ROS production. It is established that mitochondrial redox potential and membrane potential strongly influence mitochondrial ROS production; high ROS production is associated with a highly negative redox potential and a polarized mitochondrial membrane (24–28), conditions that occur when mitochondrial oxygen consumption is low. It remains unknown how altered respiration, redox potential, and mitochondrial membrane potential affect ROS production from mitochondria following ischemia. Therefore, a second objective was to determine if increased ROS production in mitochondria isolated from previously ischemic myocardium is a result of mitochondrial driving forces that were altered by ischemia. We hypothesize that altered mitochondrial redox potential and ΔΨ after ischemia predict the increase in ROS production from previously ischemic ventricular tissue.

## METHODS

### Ex Vivo Heart Preparation

Animal protocols were approved by the George Washington University’s Animal Care and Use Committee and in agreement with National Institutes of Health’s *Guide for the Care and Use of Laboratory Animals*. Male Sprague-Dawley rats (*n* = 37 total) were anesthetized with an intraperitoneal injection of telazol (40 mg/kg). Male rats were studied to limit the possible effects of sex hormones and the menstrual cycle on the results. After the cessation of pain reflexes, hearts were rapidly extracted and Langendorff perfused via the aorta at a hydrostatic pressure of 70 mmHg with a Krebs–Henseleit (KH) buffer containing (in mM) 115 NaCl, 25 NaHCO_3_, 3.3 KCl, 2.2 CaCl_2_, 1.4 MgSO_4_, 1.0 KH_2_PO_4_, 5.0 glucose, and 1.0 lactate at pH = 7.4, 37°C, and oxygenated with 95% O_2_-5% CO_2_. Excess tissue was trimmed away, and a latex balloon (size 5) was inserted into the left ventricle through the left atrium and mitral valve. The balloon was attached via a tube to a pressure transducer and spindle syringe; the diastolic left ventricular pressure was set to 10 mmHg. Isovolumic left ventricular pressure (LVP) was continuously monitored and recorded using a PowerLab unit and Lab Chart software (AD Instruments). Heart rate was measured from the LVP signal. Hearts were superfused with KH in a water-jacketed beaker to maintain the temperature at 37°C. Temperature was continuously monitored via a temperature probe and averaged at 37.1 ± 0.3°C.

Approximately every 10 min, a subset of hearts was elevated above the superfusion beaker to measure coronary flow rate (CFR) and epicardial NADH fluorescence (fNADH). CFR was measured by timed collections of coronary effluent in a graduated cylinder. A UV LED spotlight with a peak wavelength of 365 nm (Mightex Systems) driven by an LED controller (Mightex Systems) illuminated the hearts for epicardial fNADH assessments (29). Emitted light was band-pass filtered at 475 ± 25 nm and imaged using a CCD camera (Andor iXon) with a 0.5-s exposure time.

After a 10-min stabilization period following aortic cannulation, hearts were either *1*) perfused continually at a hydrostatic pressure of 70 mmHg for 60 min (control) or *2*) subjected to 60 min of global no-flow ischemia by termination of flow to the aorta (ischemia). LVP, CFR, HR, and fNADH were monitored over the 60-min experimental protocol, after which hearts underwent mitochondrial isolation.

### Mitochondrial Isolation

Immediately following the 60 min of either experimental condition, the atria and base of the heart were removed, and the remaining ventricular tissue was transferred to an ice-cold solution containing (in mM) 100 KCl, 40 Tris·HCl, 10 Tris base, 5 MgCl_2_, 1 EDTA, and 1 ATP at pH 7.4 (*solution I*) (30). The tissue was separated, minced on ice in a petri dish, weighed, and placed in 9 mL of *solution I*. Protease (subtilisin A; Sigma-Aldrich) was added (5 mg/g wet tissue), and the digested mince was mixed continually for 7 min. *Solution I* (10 mL) was added to end digestion, and the mince was homogenized with a tissue homogenizer (IKA) for 30 min at 4,000 rpm. This homogenate was centrifuged at 700 *g* at 4°C for 10 min. The supernatant of the first 700-*g* spin was stored on ice, and the pelleted material was resuspended in 19-mL *solution I* and recentrifuged at 700 *g*, and the supernatant was added to the supernatant of the first spin to increase mitochondrial yield. Respiration parameters did not differ between mitochondria isolated from the supernatants of the first and second 700-*g* spins (data not shown). The supernatant was then centrifuged at 10,000 *g* for 10 min, the pellet was resuspended in 15 mL of *solution II* containing (in mM) 100 KCl, 40 Tris·HCl, 10 Tris base, 1 MgCl_2_, 0.1 EDTA, 0.2 ATP, and 2% (wt/vol) bovine serum albumin (BSA) (A-7030, Sigma-Aldrich; fatty acid content < 0.01%) at pH 7.4. This suspension was centrifuged at 7,000 *g* for 10 min. The supernatant was then discarded, and the pellet was resuspended in a solution identical to *solution II* but without BSA (*solution III*). This resuspended pellet was subsequently centrifuged at 3,500 *g* for 10 min. The final mitochondrial pellet was suspended in 400 µL of a solution containing (in mM) 220 mannitol, 70 sucrose, 10 Tris·HCl, and 1 EGTA at pH 7.4. Protein concentration was determined by a Pierce BCA Protein Assay Kit (Thermo Fisher Scientific). Cytochrome-*a* concentration was measured optically as previously described, using 750–1,500 μg of protein (31).

### Isolated Mitochondria Measurements

#### Maximal, resting, and intermediate oxygen consumption rates with different fuels.

Mitochondrial oxygen consumption rate (*J*_O_) was measured polarographically in a respiration chamber (WPI) maintained at 37°C in respiration medium (RM), adapted from Wanders et al. (12), containing (in mM) 100 KCl, 50 MOPS, 20 glucose, 10 K_2_PO_4_, 10 MgCl_2_, 1 EGTA, and 0.2% BSA at pH 7.0. Not every experiment was conducted for each heart because of the limited mitochondrial yield resulting from 1.0 to 1.5 g of harvested ventricular tissue. In a subset of mitochondrial isolations, 100–200 μg of mitochondrial protein were incubated in 1.2-mL RM and maximal (state 3) *J*_O_ was stimulated with 1.0 mM ADP in the presence of *1*) 1 mM pyruvate (P) + 1 mM malate (M); *2*) 10 μM palmitoyl-l-carnitine (PC) + 1 mM M; *3*) 20 mM glutamate (G) + 1 mM M, or 10 mM succinate. Because this amount of ADP resulted in the utilization of all the oxygen in the respiration chamber, resting (state 4) *J*_O_ was determined before state 3 by adding 0.125 mM ADP before eliciting state 3 with 1.0 mM ADP. When succinate respiration rates were assessed, after a steady-state state 2 was achieved, 1.0 mM ADP was added; there was no assessment of state 4. The respiratory control ratio (RCR) was calculated as the state 3:state 4 ratio (32).

Intermediate respiration rates were elicited using the creatine kinase (CK) energy clamp previously described (20–22, 33), using the same fuel combinations used for state 3 respiration experiments. Briefly, after the addition of mitochondria and fuels, containing (in mM) 2.5 PCr, 5.0 ATP, and 5.0 Cr, excess creatine kinase (75 U/mL) were added to the RM. Respiration was followed and subsequent additions of PCr were made to result in final [PCr] of 5, 7.5, 10, 15, and 20 mM, PCr:Cr ratios of 0.5, 1.0, 1.5, 2.0, 3.0, and 4.0, and Gibbs-free energy of ATP hydrolysis (ΔG_ATPe_) values of −13.1, −13.5, −13.8, −14.0, −14.2, and −14.4 kcal/mol, calculated using the PCr:Cr ratio and the standard ${\text{ΔG}}_{\text{ATP}}^{{}^{\circ}}$:

$${\text{ΔG}}_{{\text{ATP}}_{\text{e}}}={\text{ ΔG}}_{\text{ATP}}^{{}^{\circ}}-2.3\;R\text{T}\;\log\;\frac{\left[\text{PCr}\right]K_{\text{CK}}}{\left[\text{Cr}\right][{\text{P}}_{\text{i}}]},$$

where ${\text{ΔG}}_{\text{ATP}}^{{}^{\circ}}$ = −7.59 kcal/mol, *R* is the gas constant (1.99 cal/K/mol), and T is the temperature (310 K).

#### Measuring NADH, ΔΨ, and J_O_ at varying values of ΔG_ATP_.

In a subset of mitochondrial isolations, intermediate respiration rates with P + M and G + M were elicited as described in the previous section, and mitochondrial membrane potential (ΔΨ) and *J*_O_ were measured simultaneously. A TPP^+^-sensitive microelectrode (KWIKTPP, WPI) was used to measure the distribution of the lipophilic cation TPP^+^ across the inner mitochondrial membrane. The microelectrode was attached to a standard pH meter, and the signal was recorded by a PowerLab unit and LabChart software (AD Instruments). TPP^+^ (4 µM final concentration) was added to the RM before the mitochondria and the TPP^+^ signal was acquired continuously while varying ΔG_ATP_, using the CK energy clamp, as described in *Maximal, resting, and intermediate oxygen consumption rates with different fuels*.

Parallel experiments were conducted in the respiration chamber with more mitochondrial protein (∼500 μg) to achieve a signal:noise ratio high enough to measure mitochondrial NADH fluorescence. A high-powered UV light source (centered at 365 nm, Mightex Systems) was connected to a bifurcated fiber optic cable (Ocean Optics) and excitation light was guided into the mitochondrial suspension. Emitted light was bandpass filtered at 550 ± 140 nm (MidOpt) and guided to a spectrometer (QEPro, Ocean Optics). Emission spectra were saved using OceanView software (Ocean Optics). In these experiments, ∼500 μg of mitochondrial protein was added to the RM (containing 10 mM P_i_), and the incubation was primed with 0.125 mM ADP in the absence of fuels to achieve maximal oxidation of NADH. The CK clamp was then performed. Incubations respired to anoxia, allowing for the determination of maximally reduced NADH.

#### Net H_2_O_2_ release.

In a subset of mitochondrial isolations, mitochondria from control and ischemia hearts were prepared on the same day, and H_2_O_2_ release was assessed using a 96-well plate reader. Net H_2_O_2_ release by intact mitochondria was assessed using Amplex Red (AR) (ThermoFisher Scientific). Mitochondria (∼25 µg) were incubated in 200-µL RM at 37°C in the presence 50 µM AR and 0.1 U/mL horseradish peroxidase as previously described (22). Fuel combinations included P + M, G + M, and G + M+ 2 mM arsenite (A). G + M + A was used to reconstruct the mitochondrial steps of the malate-aspartate shuttle by inhibiting 2-oxogluterate (2-OG) dehydrogenase, precluding 2-OG oxidation, and inhibiting glutamate dehydrogenase. This biases glutamate to transaminate with oxaloacetate to form aspartate, which can be exchanged with glutamic acid. This fuel combination was included as it has been shown to be a ROS-producing fuel combination (22, 26). H_2_O_2_ release was measured at three values of ΔG_ATP_ (−13.0, −14.0, and −14.2 kcal/mol), established using the CK energy clamp, and during ATP synthase inhibition with oligomycin.

### Data Analysis

#### Intact heart fNADH analysis.

Epicardial fNADH images were analyzed using SOLIS software (Andor Technology). Raw NADH fluorescence (rNADH) was averaged at each time point within a large region of interest on the free wall of the left ventricle. Because only hearts subjected to ischemia experienced changes in NADH, these signals were normalized from 0 to 1, where 0 was the minimum rNADH value and 1 was the maximum rNADH value for a given heart. The resulting normalized NADH (nNADH) values were calculated as nNADH = (rNADH − rNADH_min_)/(rNADH_max_ − rNADH_min_). For control flow, nNADH was calculated as percent change from the minimum rNADH, which always occurred during baseline flow, such that control nNADH = (rNADH − rNADH_min_)/rNADH_min_.

#### Driving forces and pathway conductances.

Force:flow analysis of the mitochondrial oxidative phosphorylation network was conducted as previously described (21, 23). It was assumed that flux down the oxidative phosphorylation pathway varied according to the thermodynamic driving forces acting on it, according to

$$J=L(\Delta {\text{G}}_{1}-\Delta {\text{G}}_{2}),$$

where *J* is the flux down the pathway, *L* is the phenomenological conductance of the relevant section of the pathway, and ΔG_1_ and ΔG_2_ are the driving forces acting on the front end and back end of the pathway, respectively.

The activity of the entire oxidative phosphorylation pathway was examined first by plotting oxygen consumption rate (*J*) against the thermodynamic driving force from the redox potential (ΔG_redox_) to the free energy of ATP (ΔG_ATP_) in control and ischemia mitochondria. The pathway was then divided to individually examine the activity of the ETC (ΔG_redox_ − ΔG_ΔΨ_) and ATP synthesis and transport (ΔG_ΔΨ_ − ΔG_ATP_). The free energy associated with fuel was assumed to be constant, and the conductance of fuel transport and the dehydrogenases was also determined (ΔG_fuel_ − ΔG_NADH_).

The oxidation/reduction (redox) potentials (*E*_h_) were then calculated as

$$E_{\text{h}}=E_{\text{m}}+\left(R\text{T}/nF\right)\times \text{log}\left[\left(\text{oxidized}\right)/\left(\text{reduced}\right)\right],$$

where *E*_m_ is the midpoint potential, *n* is the number of electrons transferred, and *F* is the Faraday constant (23.062 cal/mV/mol). *E*_m_ = −320 mV for NAD/NADH, and *E*_h_ = 820 mV for O_2_/H_2_O. It was assumed that NADH donates two electrons (*n*) to the ETC, 10 protons are pumped (m), and results in 2.7 ATP molecules (p). As such, stoichiometric free energies were calculated using

$$\Delta {\text{G}}_{\text{redox}}=-nF\left(\Delta E_{\text{h}}+\Delta \Psi \right)$$

$$\Delta {\text{G}}_{\Delta \Psi }=-\text{m}F\Delta \Psi$$

$$\Delta {\text{G}}_{\text{ATP}}=\text{p}\Delta {\text{G}}_{\text{ATPe}.}$$

#### Percent-free radical leak.

Because not all experiments were completed on the same mitochondria, the H_2_O_2_ production rates were paired with data from the CK clamp. Percent-free radical leak was calculated as H_2_O_2_ production/O_2_ consumption. These paired data were also used to determine force:flow relationships.

### Statistical Analysis

Changes in function during ischemia, differences in H_2_O_2_ release, and differences in percent-free radical leak between control and ischemia were determined by using a repeated-measures analysis of variance with a Tukey–Kramer test for post hoc comparisons. Differences between control and ischemia mitochondria for states 3 and 4, and RCR, the slopes of the force:flow relationships, and the percent contribution of each component of the oxidative phosphorylation pathway to flow resistance were compared using two-tailed Student’s *t* tests. A *P* value of <0.05 was considered statistically significant.

## RESULTS

Rat weight was not different between the control and ischemia groups (427.5 ± 24.4 and 446.5 ± 18.3 g), where the total number of animals was *n* = 17 for control and *n* = 20 for ischemia.

### Ischemic Conditioning of Ex Vivo Hearts

**Figure 1.**
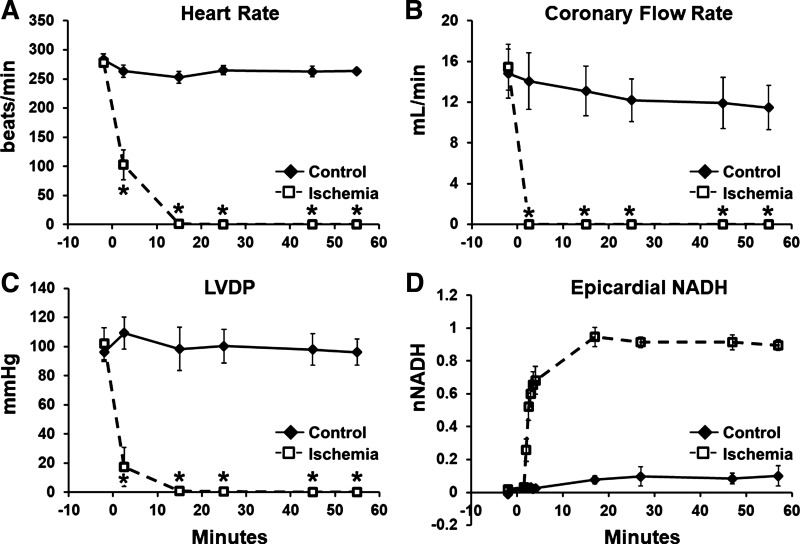
No-flow ischemia results in a lack of mitochondrial oxygenation and a decline in heart function. Heart rate (*A*), coronary flow rate (*B*), and left ventricular developed pressure (LVDP; *C*) decreased in the ischemia group when flow was terminated at *minute 0*. Epicardial NADH fluorescence (*D*) increased upon termination of flow in the ischemia group, indicating a lack of mitochondrial oxygenation, and plateaued within several minutes, indicating full reduction of NAD^+^/NADH pool for the majority of the ischemic episode. Control (♦) and ischemia (☐) are indicated; *n* = 3–7 rats per condition. Values are means ± SE. *P < 0.05, significantly different from control; repeated-measures ANOVA.

Results from ex vivo isovolumic contracting hearts are shown in Fig. 1. Heart function was not significantly different between groups during the baseline phase of perfusion, before terminating aortic flow for hearts in the ischemia group. Control and ischemia groups had no differences in baseline CFR (14.8 ± 2.4 and 15.4 ± 2.3 mL/min), HR (283.1 ± 9.8 and 278.1 ± 6.0 beats/min), or LVDP (96.2 ± 6.4 and 101.9 ± 11.0 mmHg) (Fig. 1). Upon termination of aortic flow in the ischemia group, there was an immediate decrease in CFR, HR, and LVDP. Epicardial NADH quickly rose and plateaued within several minutes (Fig. 1), indicating a complete loss of mitochondrial oxygenation and complete reduction of the NAD^+^ pool. Within 12 min, HR and LVDP had dropped to values approaching zero. In control hearts, HR, CFR, LVDP, and epicardial NADH were maintained over 60 min of perfusion (Fig. 1).

### Mitochondrial Isolations Were Similar between Control and Ischemia Hearts

Ventricular mass from which mitochondria were isolated was similar between control and ischemia groups (1.30 ± 0.07 and 1.34 ± 0.05 g). The resulting mitochondrial suspensions from control and ischemia groups were not different in protein concentrations (17.26 ± 1.87 and 15.22 ± 1.12 mg/mL) nor cytochrome-*a* content (20.06 ± 1.31 and 18.89 ± 1.07 pmol Cyt-*a*/µL). In addition, the mitochondrial yield did not differ between control and ischemia groups (5.08 ± 0.75 and 4.82 ± 0.62 mg mitochondrial protein/g ventricular tissue). The concentration of cytochrome-*a* within the suspensions was also the same between groups (1.22 ± 0.05 and 1.24 ± 0.07 nmol Cyt-*a*/mg mitochondrial protein).

### Ischemia Decreased Maximal Mitochondrial Respiration

Ischemia decreased maximal mitochondrial oxygen consumption rate for all fuel combinations assessed (Table 1). In addition to maximal (state 3) respiration, the ADP-exhausted (state 4) respiration and the respiratory control ratio (RCR) were measured. Maximal respiration decreased to less than 50% following ischemia for all substrates examined: P + M, G + M, PC + M, and succinate. Ischemia resulted in an increase in state 4 respiration with P + M and PC + M, indicating increased proton leak in these mitochondria. Finally, RCR was lower in ischemia mitochondria with all fuel combinations (Table 1).

**Table 1.** Oxygen consumption at maximal and resting respiration in control and ischemia mitochondria

	Control Mitochondria			Ischemia Mitochondria		
Substrates	State 3, nmol O_2_/nmol Cyt-*a*/min	State 4, nmol O_2_/nmol Cyt-*a*/min	RCR	State 3, nmol O_2_/nmol Cyt-*a*/min	State 4, nmol O_2_/nmol Cyt-*a*/min	RCR
G + M	450.4 ± 51.0	42.7 ± 5.0	11.1 ± 2.2	209.2 ± 24.3*	44.3 ± 8.3	5.8 ± 1.5*
P + M	551.2 ± 34.8	32.3 ± 3.1	18.1 ± 2.9	247.0 ± 34.4*	57.6 ± 6.6*	5.0 ± 1.5*
PC+M	422.5 ± 17.6	45.9 ± 1.5	9.3 ± 0.6	156.9 ± 46.6*	68.5 ± 4.5*	1.6 ± 0.2*
Succinate	209.9 ± 14.8	107.4 ± 4.9 (state 2)		91.3 ± 25.4*	63.2 ± 11.0* (state 2)	

Values are means ± SE; *n* = 3–5 rats per condition. Respiratory control ratio (RCR) = state 3/state 4. Substrates provided: pyruvate (P) = 1 mM, malate (M) = 1 mM, palmitoyl-l-carnitine (PC) = 10 µM, glutamate (G) = 20 mM, and succinate = 10 mM. State 2 values are given for succinate. Cyt-*a*, cytochrome-*a*. *P < 0.05, significantly different from control, two-tailed *t* test.

### Ischemia Impaired Intermediate Mitochondrial Respiration and Lowered *K*_mADP_

**Figure 2.**
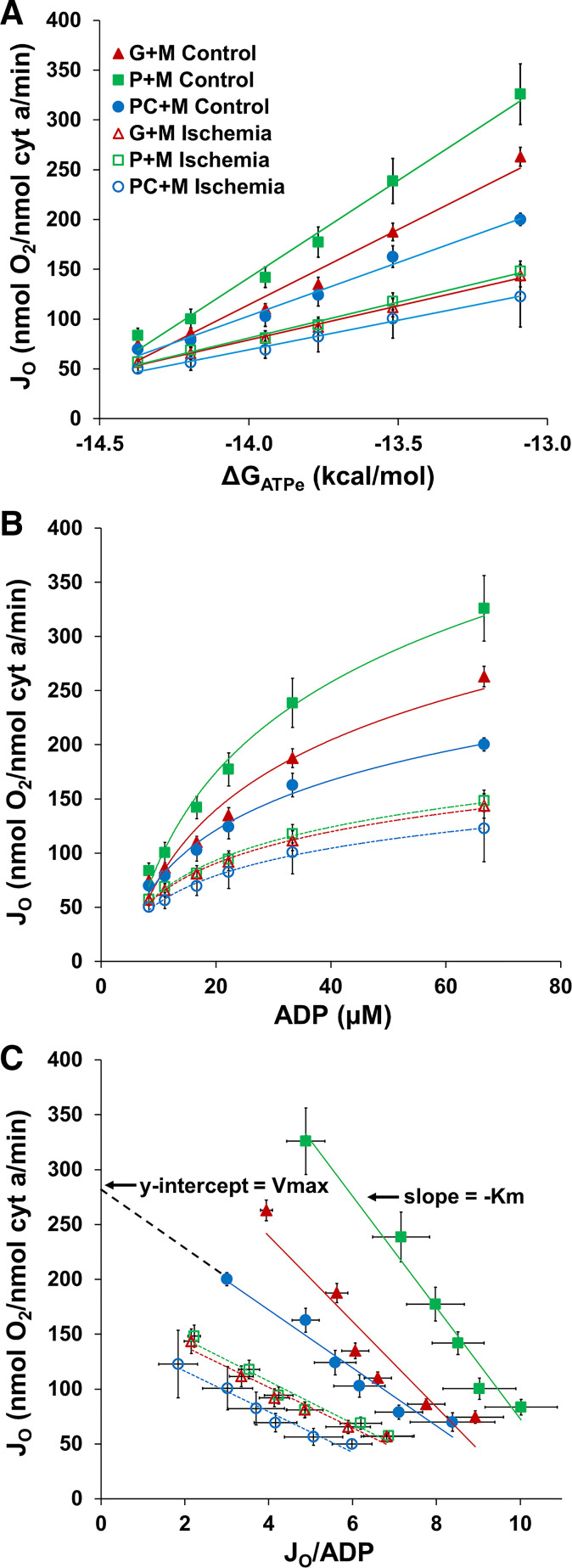
Ischemia mitochondria have lower intermediate oxygen consumption rates and *K*_mADP_ for all fuel combinations. *A*: *J*_O_ versus ΔG_ATP_ relationships are plotted for control mitochondria (closed symbols) and ischemia mitochondria (open symbols) when provided G + M (triangles), P + M (squares), or PC+M (circles). After ischemia, mitochondria had a reduced ability to increase respiration as ΔG_ATPe_ fell. *B*: Michaelis–Menten relationship between *J*_O_ and ADP in control and ischemia mitochondria demonstrated a blunted ability of ischemia mitochondria to increase respiration in response to an increase in [ADP]. *C*: Eadie–Hofstee plot for the relationship between *J*_O_ and ADP, where the *y*-intercept yields the *V*_max_ and the slope of the line is –*K*_m_. For every fuel combination, ischemia mitochondria had lower oxygen consumption rates. Values are means ± SE; *n* = 3–11 rats per condition. *K*_mADP_ and *r*^2^ values are listed in Table 2.

The progressive creatine kinase energy clamp technique was used to alter the extramitochondrial ADP:ATP ratio, and thus, the ΔG_ATP_, assessing the ability of the mitochondria to respond to changes in driving forces, as described in methods (21–23). The relationship between oxygen consumption rate and ΔG_ATPe_ was linear for both control and ischemia mitochondria (Fig. 2*A*). Control mitochondria were highly responsive to alterations in ΔG_ATPe_, shown by the steep relationship between *J*_O_ and ΔG_ATPe_ (Fig. 2*A*). Although similar respiration rates were shown in control and ischemia mitochondria at more negative values of ΔG_ATPe_ (↓ADP:ATP), ischemia mitochondria were less responsive to alterations in ΔG_ATPe_, with impaired respiration rates at less negative values of ΔG_ATPe_ (↑ADP:ATP) (Fig. 2*A*).

Eadie–Hofstee plots provided the *V*_max_ and *K*_mADP_ for control and ischemia mitochondria, calculated from the *y*-intercept and the slope (Fig. 2*C*) (22, 34, 35). *V*_max_ values calculated from the Eadie–Hofstee plots were similar to the experimentally measured state 3 maximal oxygen consumption rates (Tables 1 and 2). In addition, *K*_mADP_, or the responsiveness of mitochondrial respiration to [ADP], was lower for ischemia mitochondria for all fuels (Fig. 2*C*, Table 2). Interestingly, these data suggest ischemia mitochondria were more sensitive to a rise in extramitochondrial ADP concentration; however, the maximal respiration rate that could be achieved was reduced.

**Table 2.** V_max_ and K_mADP_ calculated from Eadie-Hofstee plots in Fig. 2

	Control Mitochondria			Ischemic Mitochondria		
Substrates	*K*_mADP_, µM	*V*_max_, nmol O_2_/nmol Cyt-*a*/min	*r* ^2^	*K*_mADP_, µM	*V*_max_, nmol O_2_/nmol Cyt-*a*/min	*r* ^2^
G + M	39.0 ± 5.6	387.4 ± 27.6	0.84 ± 0.02	24.6 ± 4.1*	205.1 ± 24.1*	0.92 ± 0.02
P + M	48.5 ± 4.1	543.1 ± 55.5	0.90 ± 0.01	21.7 ± 2.7*	202.4 ± 20.2*	0.90 ± 0.03
PC+M	33.4 ± 6.1	292.8 ± 15.9	0.93 ± 0.02	17.6 ± 4.5*	170.1 ± 38.9*	0.95 ± 0.01

Values are means ± SE; *n* = 3–11 rats per condition. Eadie–Hofstee analysis (Fig. 2*C*) revealed ischemia lowered the ADP sensitivity (*K*_mADP_) and maximal oxygen consumption (*V*_max_) for all substrates examined, consistent with the state 3 rates presented in Table 1. G, glutamate; M, malate; P, pyruvate; PC, palmitoyl-l-carnitine; Cyt-*a*, cytochrome-*a*. *P < 0.05, significantly different from control, two-tailed *t* test.

### Ischemia Diminished Responsiveness to Driving Forces and Decreased Pathway Activities

**Figure 3.**
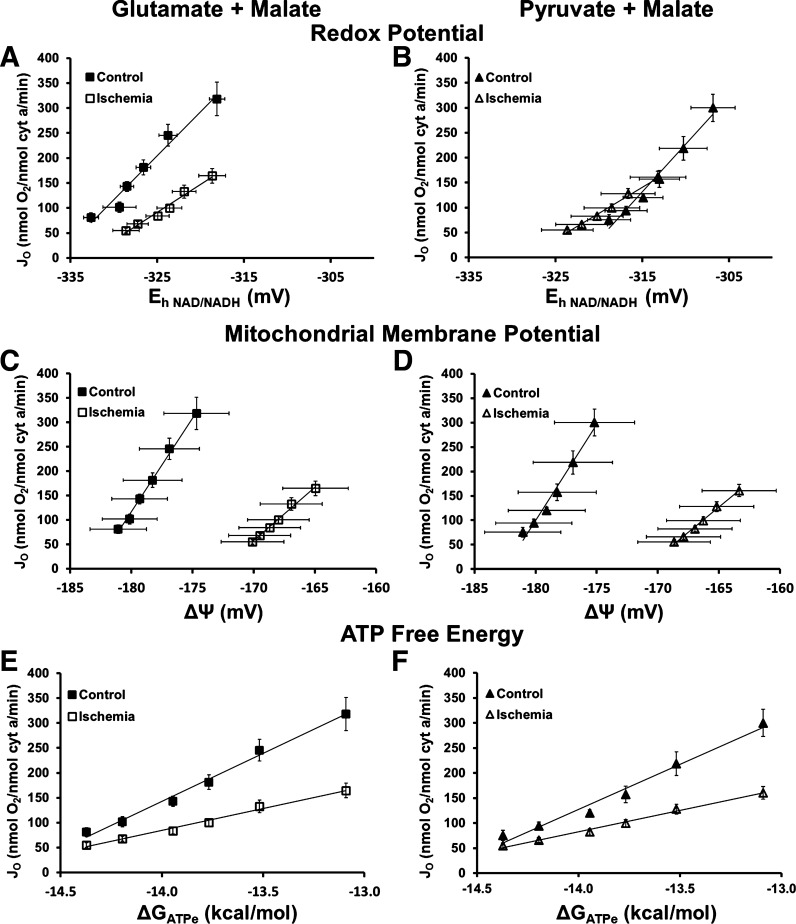
Ischemia alters the relationship between oxygen consumption rate and mitochondrial driving forces. Plots of oxygen consumption rate versus energetic driving forces (*E*_h NAD/NADH_, ΔΨ, and ΔG_ATPe_) reveal the linearity of the relationships for control (closed symbols) and ischemia (open symbols) mitochondria when provided either glutamate + malate (G + M, triangles, *left*) or pyruvate + malate (P + M, squares, *right*) as fuel. *A* and *B*: *J*_O_ versus redox potential (*E*_h NAD/NADH_) relationships for ischemia mitochondria indicate lower sensitivity to redox potential than control mitochondria. *C* and *D*: *J*_O_ versus ΔΨ relationships for ischemia mitochondria reveal a lower range of oxygen consumption rates and less negative mitochondrial membrane potentials. *E* and *F*: *J*_O_ versus ΔG_ATPe_ relationships indicate that ischemia mitochondria have a much lower slope (i.e., conductance) than control mitochondria. Values are means ± SE; *n* = 4–12 rats per condition. Slopes (*E*_h NAD/NADH_, ΔΨ, and ΔG_ATPe_) and *r*^2^ values are listed in Table 2.

Mitochondrial driving forces (redox potential (*E*_h NAD/NADH_), mitochondrial membrane potential (ΔΨ), and ΔG_ATPe_) were determined in control and ischemia mitochondria fueled with G + M and P + M. The relationships between mitochondrial oxygen consumption rate and each of the mitochondrial driving forces (*E*_h NAD/NADH_, ΔΨ, and ΔG_ATP_) are shown in Fig. 3. The slope of each of these relationships is an indication of how responsive oxygen consumption is to each specific driving force. In all cases, ischemia mitochondria demonstrated lower responsiveness (lower slope) to the energetic driving forces compared with control (Fig. 3, Table 3).

**Table 3.** Mean slopes for the force-flow relationships in Fig. 3

	Figure	Fuel	Control Mitochondria		Ischemia Mitochondria	
			Slope	*r* ^2^	Slope	*r* ^2^
*E*_h NAD/NADH_, mV	3*A*	G + M	21.0 ± 1.3	0.93 ± 0.03	10.3 ± 1.5*	0.97 ± 0.01
	3*B*	P + M	18.0 ± 3.7	0.91 ± 0.08	10.4 ± 1.7*	0.94 ± 0.06
ΔΨ, mV	3*C*	G + M	39.9 ± 4.4	0.97 ± 0.01	22.8 ± 2.7*	0.96 ± 0.01
	3*D*	P + M	43.2 ± 5.6	0.98 ± 0.01	26.0 ± 4.0*	0.96 ± 0.01
ΔG_ATPe_, kcal/mol	3*E*	G + M	180.0 ± 19.4	0.97 ± 0.01	84.4 ± 9.8*	0.98 ± 0.01
	3*F*	P + M	192.1 ± 21.4	0.98 ± 0.00	87.8 ± 12.9*	0.97 ± 0.01

Values are means ± SE; *n* = 4–12 rats per condition. The slopes of the *E*_h NAD/NADH_: *J*_O_, ΔΨ:*J*_O_, and ΔG_ATPe_: *J*_O_ relationships (Fig. 3, *A* and *B, C* and *D*, and *E* and *F*, respectively) were lower in mitochondria following ischemia, demonstrating a blunted sensitivity to mitochondrial driving forces following ischemia. G, glutamate; M, malate; P, pyruvate. *P < 0.05, significantly different from control, two-tailed *t* test.

**Figure 4.**
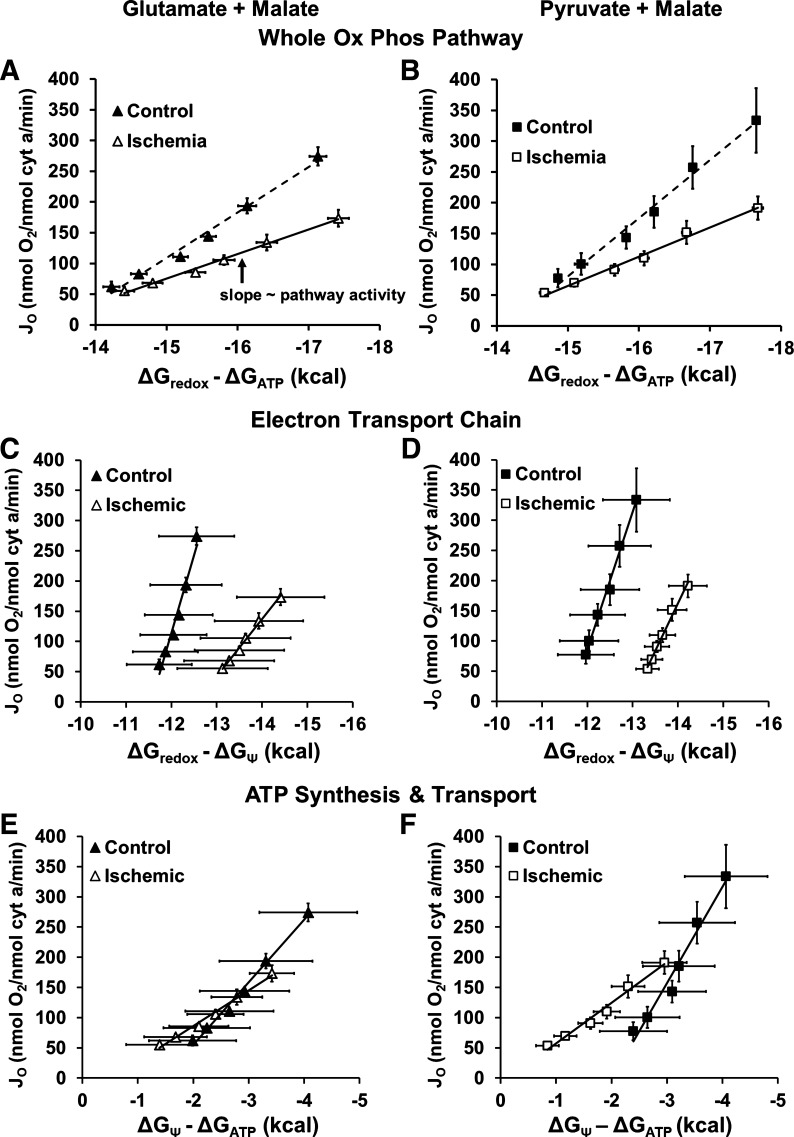
Ischemia reduces the conductance of the oxidative phosphorylation pathway. Force:flow relationships are plotted for control (closed symbols) and ischemia (open symbols) mitochondria when provided with glutamate + malate (G + M, triangles, *left*) or pyruvate + malate (P + M, squares, *right*). The slope of the force:flow relationship represents the conductance of that component of oxidative phosphorylation. *A* and *B*: force:flow relationship of *J*_O_ and the difference in free energy between redox potential (*E*_h NAD/NADH_) and ΔG_ATP_, representing the conductance of the entire oxidative phosphorylation pathway (*L*_OxPhos_), which is reduced in ischemia mitochondria. *C* and *D*: force:flow relationship between *J*_O_ and the difference in free energy between redox potential and ΔΨ represents the conductance of the electron transport chain (*L*_ETC_). The lower slope in ischemia mitochondria indicates a decreased conductance, or effective activity of the ETC, following ischemia. *E* and *F*: force:flow relationship between *J*_O_ and the difference in free energy between ΔΨ and ΔG_ATP_ represents the conductance of the combined activity of the ATP synthase and the adenine nucleotide translocase (ANT) (*L*_ATP synth +ANT_). Ischemia decreases the activity of this component, which is responsible for ATP synthesis and transport from the mitochondrial matrix to the intermembrane space. Data are means ± SE; *n* = 4–6 rats per condition. Slopes (*L*_OxPhos_, *L*_ATP synth +ANT_, *L*_ETC_) and *r*^2^ values are listed in Table 2.

Flux through the mitochondrial oxidative phosphorylation pathway is a function of *1*) the effective activity (conductance) of the pathway and *2*) the driving force across the pathway. To determine the entire oxidative phosphorylation pathway activity, the potential energy drop from the mitochondrial redox potential (ΔG_redox_) to the free energy of ATP (ΔG_ATP_) was plotted against oxygen consumption rate, as previously described (21, 23). The slope of this relationship indicates the responsiveness of the pathway, with a steeper slope indicating a greater conductance, or effective activity, of that pathway (Fig. 4, *A* and *B*). These data show that ischemia resulted in a decrease in the conductance of the entire mitochondrial oxidative phosphorylation cascade: 2.4- and 2.0-fold with G + M and P + M, respectively (Table 4).

**Table 4.** Mean slopes for the force-flow relationships in Fig. 4

	Figure	Fuel	Control Mitochondria		Ischemia Mitochondria	
			Slope	*r* ^2^	Slope	*r* ^2^
*L*_OxPhos_	4*A*	G + M	74.2 ± 7.8	0.98 ± 0.01	30.4 ± 2.2*	0.98 ± 0.01
	4*B*	P + M	93.5 ± 11.3	0.98 ± 0.01	42.6 ± 7.0*	0.97 ± 0.01
*L*_fuel/DH_	3*A*	G + M	401.4 ± 58.4	0.93 ± 0.03	195.3 ± 34.2*	0.97 ± 0.01
	3*B*	P + M	394.0 ± 60.9	0.91 ± 0.05	303.6 ± 79.0*	0.97 ± 0.01
*L*_ETC_	4*C*	G + M	275.9 ± 50.2	0.94 ± 0.04	107.4 ± 19.2*	0.97 ± 0.02
	4*D*	P + M	252.2 ± 73.0	0.94 ± 0.02	142.9 ± 39.8*	0.93 ± 0.04
*L*_ATP synth + ANT_	4*E*	G + M	109.3 ± 19.4	0.98 ± 0.01	40.7 ± 3.7*	0.98 ± 0.01
	4*F*	P + M	166.9 ± 29.0	0.94 ± 0.02	55.7 ± 14.6*	0.96 ± 0.02
*L*_H2O2_	6*E*	G + M	70.4 ± 22.2	0.88 ± 0.04	72.3 ± 20.0	0.90 ± 0.05
	6*F*	P + M	28.4 ± 7.2	0.90 ± 0.80	45.2 ± 15.4	0.89 ± 0.08

Values are means ± SE; *n* = 4–6 rats per condition. Control of mitochondrial respiration was altered after ischemia, with ischemia mitochondria demonstrating a ubiquitous reduced activity across the oxidative phosphorylation pathway. Ischemia decreased the slope, or the pathway activity, of *1*) the fuel dehydrogenases, *2*) the electron transport chain, and *3*) ATP synthase and the adenine nucleotide translocase. However, the slope of the ΔG_redox_ − ΔG_Ψ_:H_2_O_2_ relationship was unchanged after ischemia, indicating that a given change in driving force would result in the same increase in H_2_O_2_ release in both control and ischemia mitochondria. G, glutamate; M, malate; P, pyruvate. *P < 0.05, significantly different from control, two-tailed *t* test.

The activity of *1*) the ETC and *2*) the combined activities of ATP synthase/ANT/P_i_ transporter (ATP synthesis and transport) were next examined individually by plotting the driving force across the ETC (ΔG_redox_ − ΔG_ΔΨ_) and the driving force across ATP synthase and ANT (ΔG_ΔΨ_ − ΔG_ATP_) against respiration rate (Fig. 4, *C*–*F*). Ischemia resulted in a decrease in the effective activity of both the ETC (Fig. 4, *A* and *B*) and ATP synthesis and transport (Fig. 4, *C* and *D*). Taken together, these data indicate that ischemia resulted in a decrease in the activity of the entire oxidative phosphorylation pathway that was caused by decreases in the activities of both the ETC and ATP synthesis and transport.

Finally, the relative resistance of each component of the oxidative phosphorylation cascade was determined to elucidate the distribution of control across the oxidative phosphorylation pathway. To do this, the resistances (i.e., 1/conductance) of each of the pathways to energy transfer were determined under control and ischemia conditions. The total resistance of the oxidative phosphorylation pathway was determined by summing the resistances to energy transfer of the individual pathway components, i.e., *1*) fuel transport and the dehydrogenase enzymes, *2*) the ETC, and *3*) ATP synthesis and transport. The contribution of each individual component to resistance was then determined (21).

Although ischemia lowered the activity of the entire oxidative phosphorylation pathway, it did not alter the distribution of control in the oxidative phosphorylation pathway. In control mitochondria, fuel transport and the dehydrogenase enzymes contributed ∼15%, the ETC contributed ∼25%, and ATP synthesis and transport contributed ∼60% to the total pathway resistance (Table 5). This distribution was not significantly different after ischemia.

**Table 5.** Contribution of each oxidative phosphorylation pathway component to the total resistance to energy transfer

	Fuel Transport/Dehydrogenases, %	Electron Transport Chain, %	ATP Production/Transport, %
G + M			
Control	16.4 ± 2.2	24.5 ± 4.7	59.1 ± 5.4
Ischemia	13.4 ± 1.6	25.2 ± 4.6	61.4 ± 4.5
P + M			
Control	19.0 ± 0.9	34.0 ± 7.0	47.1 ± 7.1
Ischemia	15.6 ± 5.4	28.9 ± 5.5	69.9 ± 10.5

Values are means ± SE; *n* = 4–6 rats per condition. Ischemia lowered the activity of every segment of the oxidative phosphorylation pathway but did not alter the percent contribution of each component of the total resistance of the pathway. G, glutamate; M, malate; P, pyruvate. *P < 0.05, significantly different from control, two-tailed *t* test.

### H_2_O_2_ Release Increased following Ischemia and is Related to Lower ETC Effective Activity

**Figure 5.**
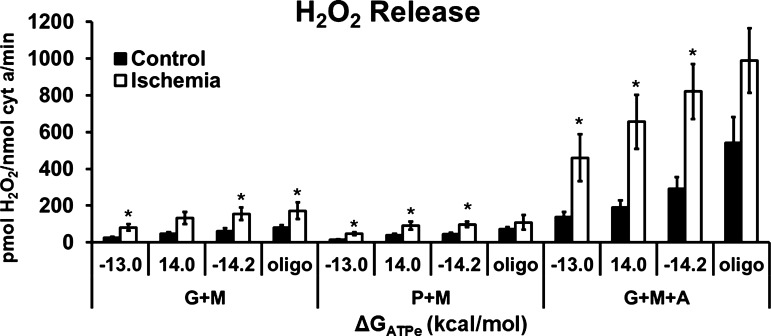
H_2_O_2_ production was higher in ischemia compared with control mitochondria. Net H_2_O_2_ release from mitochondria respiring at ΔG_ATPe_ = −13.0, −14.0, −14.2 kcal/mol, and under oligomycin conditions when provided glutamate + malate (G + M) and pyruvate + malate (G + M). The mitochondrial steps of the malate–aspartate shuttle were also reconstructed using G + M+arsenite (G + M+A) to examine a condition of high H_2_O_2_ release. Data are means ± SE; *n* = 5–7 rats per condition. *P < 0.05, significantly different from control; repeated-measures ANOVA.

**Figure 6.**
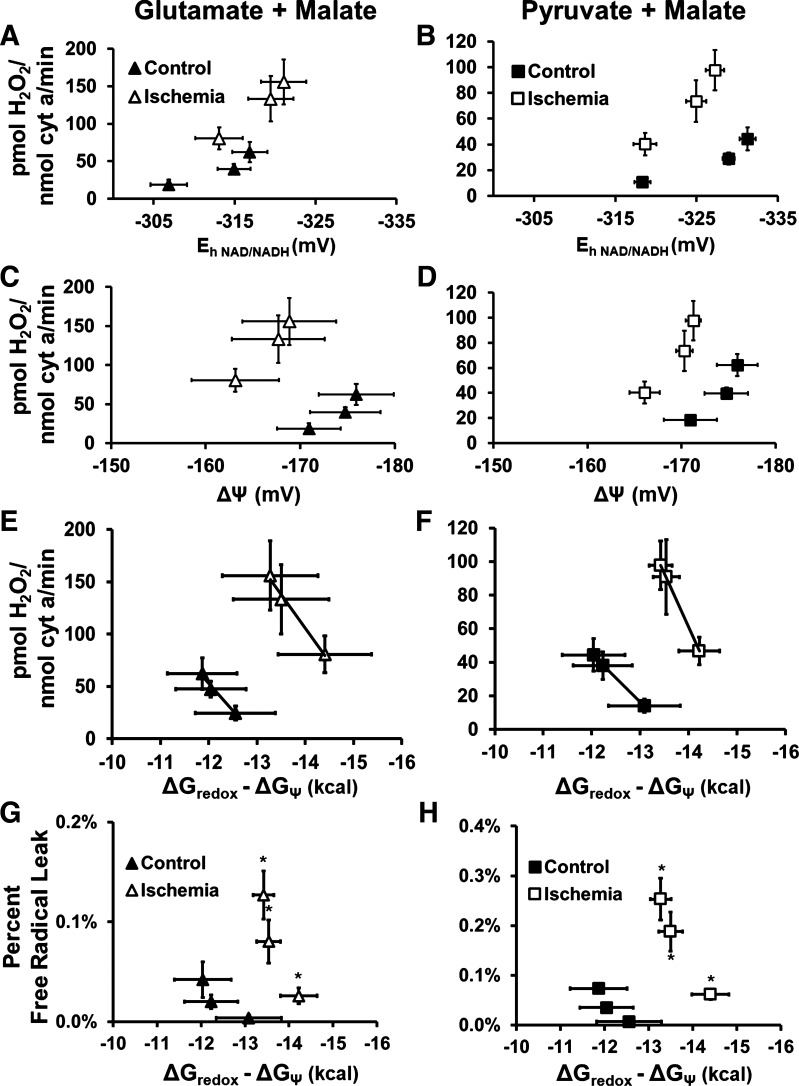
H_2_O_2_ production was related to energetic driving forces in both control and ischemia mitochondria. H_2_O_2_ production increased in both control (closed symbols) and ischemia (open symbols) mitochondria with increasing NADH reduction (*A* and *B*) and a more negative ΔΨ (*C* and *D*). Because both redox potential and mitochondrial membrane potential predict electron leak from the mitochondria, the H_2_O_2_ release was plotted against driving force down the ETC (ΔG_redox_ − ΔG_Ψ_; *E* and *F*), and the slopes of these relationships were not different between control and ischemia mitochondria (Table 4). However, at any given value of ΔG_redox_− ΔG_Ψ_, ischemia mitochondria demonstrated a higher H_2_O_2_ release. Percent-free radical leak was determined by dividing H_2_O_2_ production rate by the oxygen consumption rate (*G* and *H*), further augmenting the difference between the degree of electron leak in control compared with ischemia mitochondria. Values are means ± SE; *n* = 4–7 rats per condition. Slopes (*L*_H2O2_; *E* and *F*) and *r*^2^ values are listed in Table 4. *G* and *H*: *P < 0.05, significantly different from control; repeated-measures ANOVA.

In addition to assessing mitochondrial respiration and driving forces at intermediate rates of oxygen consumption, net H_2_O_2_ release was also measured from mitochondria at 3 values of ΔG_ATPe_: −13.0, −14.0, and −14.2 kcal/mol, resulting in three intermediate respiration rates (see Fig. 3, *E* and *F*), and in the presence of oligomycin (Fig. 5). For all fuels, P + M, G + M, and G + M + A, which reconstructed the mitochondrial steps of the malate-aspartate shuttle and has been reported as a high ROS-producing condition (22, 26), ischemia resulted in an increase in H_2_O_2_ release (Fig. 5), which was correlated with mitochondrial redox state and membrane potential (Fig. 6, *A*–*D*). In control and ischemia mitochondria, more negative potentials resulted in increases in H_2_O_2_ release, in agreement with findings from previous studies (24, 25). It is interesting to note that ischemia mitochondria demonstrated increased H_2_O_2_ release even when matched for the reduction levels of *E*_h NAD/NADH_ and ΔΨ of control mitochondria (Fig. 6, *A*–*D*).

To determine how ROS production was affected by the overall driving force across the ETC, H_2_O_2_ production was plotted against the energetic difference between redox potential and mitochondrial membrane potential (Fig. 6, *E* and *F*). Ischemia mitochondria demonstrated a greater driving force down the ETC than control, as well as higher levels of H_2_O_2_ release, indicating this greater driving force from complex I to complex IV might drive higher ROS production. Of note, the slope of this relationship did not differ between control and ischemia mitochondria (Fig. 6, *E* and *F*, and Table 4).

## DISCUSSION

Our analyses of mitochondrial respiration rates and driving forces using the creatine kinase energy clamp and two substrate combinations have provided new insights into the consequences of myocardial ischemia on mitochondrial respiration and ROS production. Ischemia reduced maximal and intermediate respiration rates and diminished mitochondrial membrane potential. A force:flow analysis of each segment of the energy conversion cascade revealed that depressed respiration rates were a result of decreased activities of each component of the mitochondrial energy conversion cascade: the dehydrogenase enzymes, the electron transport chain (ETC), and ATP synthesis and transport (ATP synthase/ANT/P_i_ transporter). Interestingly, ATP synthesis and transport exhibited the highest resistance within the energy cascade for both normoxic and ischemic mitochondria. Ischemia also increased ROS production for all substrate combinations, with previously ischemic mitochondria demonstrating a greater propensity to leak electrons that were not predicted directly by redox potential or mitochondrial membrane potential; instead, electron leak was likely increased because of altered driving force down the ETC or a decrease in the effective activity of the ETC.

### Control of Respiration in Control and Ischemia Mitochondria

It is generally accepted that the control of mitochondrial respiration is distributed throughout the oxidative phosphorylation cascade, with no single rate-limiting step (9–13). To determine how an ischemic insult alters the distribution of respiratory control, we examined the sensitivity of respiration to the individual mitochondrial driving forces: redox potential, membrane potential, and ΔG_ATP_ by chemically altering ΔG_ATP_ in the media and recording the resultant respiration rate (Fig. 3). In both control and ischemia mitochondria, respiration rate increased linearly as driving forces decreased; however, ischemia mitochondria were less sensitive to these driving forces and showed a blunted ability to increase respiration rate in response to a change in the mitochondrial driving forces (Fig. 3, *A*–*F*, and Table 3). This indicates that when myocardial ATP demand increases, mitochondria that previously experienced ischemia will have a reduced ability to respond to cellular signals to increase oxygen consumption and ATP production. It is also interesting to note that ischemia mitochondria exhibit diminished membrane potentials, consistent with previous investigations (14, 36), which would result in a decreased driving force for ATP synthesis; however, ATP production was not directly measured in the present study.

Although oxygen consumption was linearly related to each independent mitochondrial driving force (redox potential, membrane potential, and ΔG_ATP_), this analysis only assesses the sensitivity of respiration to an individual driving force and does not identify which pathway activities are altered. For example, ETC flux can be increased in one of three ways: *1*) by increasing the (upstream) redox potential driving force, *2*) by decreasing the (downstream) driving force of the mitochondrial membrane potential, or *3*) by increasing the effective activity of the ETC itself. Thus, to determine the combined and individual pathway activities, independent of the driving forces, oxygen consumption rates were plotted against *1*) the driving force down the entire oxidative phosphorylation pathway (Fig. 4, *A* and *B*), *2*) the driving force down the ETC (Fig. 4, *C* and *D*), and *3*) the driving force down the ATP synthesis and transport pathway (Fig. 4, *E* and *F*). The conductance, or effective activity of each pathway, was then computed as the slope of each oxygen consumption versus driving force relationship, with a greater slope representing increased pathway activity. We found that ischemia decreases the conductance of all pathways within the oxidative phosphorylation cascade, from redox potential to ΔG_ATP_. This decrease in pathway activity is a result of both a decrease in the conductance of the ETC and the ATP synthesis and transport system (Fig. 4, *C*–*F*).

Previous studies of isolated mitochondria used metabolic control analysis to quantify the control that a specific pathway component may have in modulating oxidative phosphorylation flux (37). However, to do so, inhibitors are typically titrated into a mitochondrial suspension to decrease the activity of a given component, and the ultimate change in flux is then measured (37). Such experiments demonstrate that control of oxidative phosphorylation is distributed throughout the oxidative phosphorylation cascade during intermediate mitochondrial respiration rates (9, 13), with complex IV, complex V, and ANT important locations of control (14, 38, 39). Using the approach of Glancy et al. (21), we calculated the distribution of control between the fuel transporters and dehydrogenase enzymes, the ETC, and ATP synthesis and transport (ATP synthase/ANT/P_i_ transporter), during intermediate respiration rates without the need for inhibitors. We found that all components within the oxidative phosphorylation cascade contributed to the control of oxygen consumption, in a manner that is analogous to similarly resistive electrical components connected in series.

In previous normoxic mitochondria, the fuel transporters and dehydrogenase enzymes provide ∼15% of energy transfer control, the ETC provides ∼25%, and ATP synthesis and transport (ATP synthase/ANT/P_i_ transporter) provides the highest at ∼60% (Table 5). This distribution is consistent with the inhibitor titration data of earlier studies, underscoring an important role of ANT in the control of oxidative phosphorylation during intermediate respiration in cardiac mitochondria (16, 38). It is interesting to note that this distribution differs from that of skeletal muscle mitochondria, where the fuel transporters and dehydrogenase enzymes exert ∼15% of energy transfer control, the ETC accounts for ∼60%, and the ATP synthase and ANT contribute ∼25% (21). Many reports implicate the ETC or proton leak (14, 18, 19, 40, 41) as the site of damage during ischemia, whereas others indicate ANT (16) as the primary cause of reduced respiration rates following ischemia. In the present study, after 60 min of myocardial ischemia, the conductance of the fuel transporters and dehydrogenase enzymes, the ETC, and ATP synthesis and transport all decrease (Fig. 4, Table 3). Moreover, we found that the conductance of each pathway component decreases equally by ∼50% (see the slopes in Table 3). Because the conductance of the entire energy pathway also decreases by 50%, there is a similar distribution of energetic control after ischemia, where the fuel transporters and dehydrogenase enzymes still provide ∼15%, the ETC still contribute ∼25%, and ATP synthesis and transport still contribute ∼60% of energy transfer control. Altogether, these new findings indicate that the ATP synthase and ANT pathway is the site of lowest conductance, and therefore the site of greatest energetic control, within the entire mitochondrial energy cascade, and that an episode of myocardial ischemia does not alter the relative energetic control of ATP synthesis and transport.

### Control of ROS Production in Control and Ischemia Mitochondria

Conditions that promote maximal ROS generation from the ETC have been established in many species and tissues and include low ETC electron flux, hyperpolarized mitochondrial membrane potential, and a highly reduced mitochondrial redox state (24–28). In healthy mitochondria, changes in ETC electron flux, membrane potential, and redox state all occur in synchrony during mitochondrial respiration. The process is initiated by an increase in ADP:ATP ratio, which stimulates flux through ATP synthase and ANT, which dissipates mitochondrial membrane potential and increases ETC flux, which dissipates mitochondrial redox potential (26). If unimpeded, ROS production during this elegant process is low due to low mitochondrial driving forces and high mitochondrial oxygen consumption rates, outcomes that are confirmed by our results (Fig. 6). Our results further confirm that a less reduced redox potential and less negative mitochondrial membrane potential are associated with lower ROS production in both control and ischemia mitochondria (Fig. 6, *A*–*D*).

As expected, and supporting previous findings, mitochondrial ROS production was higher after ischemia (Fig. 5) (7, 8, 18, 19). We hypothesized that this higher ROS production was primarily due to differences in driving forces after ischemia. When examining ROS production as a function of redox or mitochondrial membrane potential, it is clear that ischemia mitochondria produce more ROS, even at values of ΔG_redox_ that were similar to those of control mitochondria (Fig. 6, *A* and *B*). Indeed, despite less negative values of ΔΨ in ischemia mitochondria, ROS production was higher (Fig. 6, *C* and *D*). However, as described in *Control of Respiration in Control and Ischemia Mitochondria*, the net driving force down the ETC is a function of both the upstream (ΔG_redox_) and downstream (ΔΨ) driving forces for ROS production. Therefore, we predicted that a lower net driving force down the ETC was responsible for higher ROS production following ischemia, but the data did not support this hypothesis (Fig. 6, *E* and *F*). Instead, driving force down the ETC increased after ischemia, primarily driven by lower mitochondrial membrane potential. This increased driving force was, in fact, associated with an increase in ROS production, contrary to our hypothesis (Fig. 6, *E* and *F*). Furthermore, it is likely that the decrease in ETC conductance from complex I to complex IV that was observed in the respiration experiments (Fig. 4, *C* and *D*) also contributed to the higher ROS production in the ischemia mitochondria. Interestingly, the slope of the ΔG_redox_-ΔΨ H_2_O_2_ relationships is the same between control and ischemia mitochondria. In other words, within a given mitochondrial “type”—previously normoxic or previously ischemic—the ROS conductance (the change in ROS production with a change in driving force down the ETC) is the same.

Cardiac mitochondria can degrade H_2_O_2_ via the glutathione (GSH), thioredoxin (TRX), and catalase pathways (42–44), and, therefore, altered activity of these pathways could explain the difference in ROS production between control and ischemia mitochondria. In the present student, ROS production was assessed as H_2_O_2_ emission from mitochondria, which could be a function of increased H_2_O_2_ production and/or decreased H_2_O_2_ clearance. The capacity of these pathways should be investigated to determine the effect of ischemia on the H_2_O_2_ degenerating pathways within mitochondria. Interestingly, energizing isolated mitochondria with fuel increases the antioxidant capacity of the TRX system, and the H_2_O_2_ degenerating system does not seem to be dependent on redox potential (45). It is possible that the decreased dehydrogenase effective activity (Table 5) could alter the ability of the TRX system to degenerate H_2_O_2_.

In summary, following ischemia, mitochondria exhibit a greater propensity to leak electrons from the ETC, which is a result of both a greater driving force down the ETC and a decreased effective activity (conductance) of the ETC from complex I to complex IV. It is possible that these differences in H_2_O_2_ emission could be a result of altered capacities of the GSH, TRX, and catalase pathways to degenerate H_2_O_2_ before it leaves the mitochondrial matrix. These changes occurred in the absence of reperfusion and would set the stage for a large burst of ROS production upon reperfusion.

### Limitations

Limitations of the current study include those of retrograde perfused excised hearts and the limited mitochondrial oxygenation provided by crystalloid perfusate, such that hearts did not experience normal in vivo metabolic conditions of normoxia and ischemia before mitochondria were isolated (29, 46–48). In addition, only male rats were examined in the present study, despite data indicating female hearts are protected from ischemia-reperfusion damage compared with male hearts (49–51). Some investigations have revealed functional differences between male and female cardiac mitochondria (52–54), particularly in terms of calcium handling (52, 53), and therefore, future studies should expand the whole pathway analysis employed in the present study to mitochondria from female hearts, both with and without ischemic damage.

Other limitations are associated with the assessment of function using mitochondria isolated from their native intracellular environment, thereby removing the influence of extramitochondrial factors on mitochondrial function, such as ischemic dysregulation of ion gradients (55). Additionally, degradation of components of the oxidative phosphorylation pathway could have occurred during 60 min of ischemia; however, cytochrome oxidase does not appear degraded based on cytochrome-*a* measurements, suggesting that major degradation of energy conversion pathway components is not primary driver of these results. In addition, the creatine kinase energetic clamp analysis of mitochondrial driving forces and energetic flows did not include an assessment of proton leak. Although state 4 respiration was significantly higher after ischemia with two-thirds of the substrate combinations examined, it accounted for <70 nmol O_2_/mg/min, under conditions when proton leak was highest, the intermediate respiration rates that were examined would result in lower proton leak than state 3 respiration, such that overall measurement artifact due to proton leak is unlikely to alter the conclusions of the respiration analysis. Finally, although lower mitochondrial membrane potentials would indicate decreased ATP production capabilities, we did not directly measure ATP production from the isolated mitochondria.

### Conclusions

Ischemia alters mitochondrial driving forces and dramatically impacts the capacity of isolated mitochondria to consume oxygen and produce ATP. The present study demonstrates that 60 min of myocardial ischemia lowers the activity of the entire oxidative phosphorylation pathway, including decreased conductance of both the electron transport chain pathway and ATP synthesis and transport pathway. An increased driving force down the ETC and a decreased conductance of the ETC were associated with greater ROS production in both control and ischemia mitochondria. These findings indicate that ischemic damage is distributed throughout the mitochondrial oxidative phosphorylation cascade and that strategies to improve mitochondrial function following ischemia should target the entire organelle in its function as the primary controller of myocyte respiration. Strategies to mitigate ROS production could either target the ETC itself to increase the effective activity or strive to modulate mitochondrial driving forces to reduce the driving force down the ETC.

## GRANTS

This work was supported by American Heart Association Postdoctoral Fellowship Award 14POST20490181 and Career Development Award 16SDG30770015 (to S.K-G.); National Heart, Lung, and Blood Institute (NHLBI) and National Institute of Arthritis and Musculoskeletal and Skin Diseases funds (to B.G.), and NHLBI Grants R01HL146169 and R01HL147279 (to M.W.K.).

## DISCLOSURES

No conflicts of interest, financial or otherwise, are declared by the authors.

## AUTHOR CONTRIBUTIONS

S.K-G. conceived and designed research; S.K-G. performed experiments; S.K-G. analyzed data; S.K-G., B.G., and M.W.K. interpreted results of experiments; S.K-G. prepared figures; S.K-G. drafted manuscript; S.K-G., B.G., and M.W.K. edited and revised manuscript; S.K-G., B.G., and M.W.K. approved final version of manuscript.
